# Preparation of Poly-(Methyl vinyl ether-co-maleic Anhydride) Nanoparticles by Solution-Enhanced Dispersion by Supercritical CO_2_

**DOI:** 10.3390/ma5101841

**Published:** 2012-10-10

**Authors:** Ai-Zheng Chen, Guang-Ya Wang, Shi-Bin Wang, Jian-Gang Feng, Yuan-Gang Liu, Yong-Qiang Kang

**Affiliations:** 1 College of Chemical Engineering, Huaqiao University, Xiamen 361021, China; E-Mail: scubme@163.com (G.-Y.W.); biomat@126.com (J.-G.F.); ygliu@hqu.edu.cn (Y.-G.L.); hqukyq@126.com (Y.-Q.K.); 2 Institute of Pharmaceutical Engineering, Institute of Biomaterials and Tissue Engineering, Huaqiao University, Xiamen 361021, China

**Keywords:** nanoparticles, nonsolvent, PVM/MA, supercritical fluids

## Abstract

The supercritical CO_2_-based technologies have been widely used in the formation of drug and/or polymer particles for biomedical applications. In this study, nanoparticles of poly-(methyl vinyl ether-co-maleic anhydride) (PVM/MA) were successfully fabricated by a process of solution-enhanced dispersion by supercritical CO_2_ (SEDS). A 2^3^ factorial experiment was designed to investigate and identify the significance of the processing parameters (concentration, flow and solvent/nonsolvent) for the surface morphology, particle size, and particle size distribution of the products. The effect of the concentration of PVM/MA was found to be dominant in the results regarding particle size. Decreasing the initial solution concentration of PVM/MA decreased the particle size significantly. After optimization, the resulting PVM/MA nanoparticles exhibited a good spherical shape, a smooth surface, and a narrow particle size distribution. Fourier transform infrared spectroscopy (FTIR) spectra demonstrated that the chemical composition of PVM/MA was not altered during the SEDS process and that the SEDS process was therefore a typical physical process. The absolute value of zeta potential of the obtained PVM/MA nanoparticles was larger than 40 mV, indicating the samples’ stability in aqueous suspension. Analysis of thermogravimetry-differential scanning calorimetry (TG-DSC) revealed that the effect of the SEDS process on the thermostability of PVM/MA was negligible. The results of gas chromatography (GC) analysis confirmed that the SEDS process could efficiently remove the organic residue.

## 1. Introduction

In the past several decades, drug-loaded microspheres created by incorporating pharmaceutical agents into biodegradable polymers have aroused increasing interest [1,2,3]. This strategy can combine protecting active compounds and releasing drugs to specific tissues at a therapeutically optimal rate [4]. The potentially utilized polymers should possess an appropriate chemical composition and molecular weight to guarantee their biodegradability, low toxicity, and ideal bonding capacity with biologically active compounds [5]. The most frequently studied polymers that meet these requirements can be represented by chitosan [6] and several poly(esters), including poly(lactic acid) (PLA) [7], poly(glycolic acid) (PGA) [8], and poly(lactic-co-glycolic acid) (PLGA) [9]. 

Among various biodegradable polymers, poly-(methyl vinyl ether-co-maleic anhydride) (PVM/MA) is a typical one with great potential in biomedical application [10,11,12]. It is a polyanhydride and can be employed as an ideal co-polymer for the fabrication of particulate dosage forms due to its bioadhesive and mucoadhesive properties [13]. Actually, when PVM/MA is hydrolyzed, each cleaved anhydride bond can generate two carboxylic groups, which can promote the formation of hydrogen bonds between polymers and mucosal components [14]; furthermore, the hydrolyzation of PVM/MA can be affected by pH and thus be pH responsive to some degree. In the last few decades, PVM/MA has been widely used for pharmaceutical purposes, as a denture adhesive or adjuvant for transdermal patches [15,16,17]. However, few studies have reported the application of PVM/MA in fabricating drug-loaded polymer particles. 

A wide range of pharmaceutical agents exploit biodegradable polymers to provide targeted delivery of drugs and control the drug release rate; nevertheless, most drug carrier system preparation strategies depend on either a high temperature process, such as spray drying [18], or organic solvent evaporation-based techniques [19] to incorporate pharmaceutical candidates into polymers and form composite microspheres. High temperature processes are not suitable for temperature-sensitive compounds, and in an organic solvent-based process, complete removal of the solvent in a subsequent procedure is tedious and rather challenging.

Over the past few decades, supercritical CO_2_-based techniques have been fully developed and widely utilized in particle engineering, especially in biomedical domains [20]. Compared with those conventional methods mentioned above, the unique advantages of supercritical CO_2_-based techniques range from mild critical points (Tc = 304.1 K, Pc = 7.38 MPa), non-toxicity, non-flammability, and no organic residue, to a relatively low price [21]. Particle preparation methodologies based on supercritical CO_2_ have involved a number of related methods from a rapid expansion of supercritical solution (RESS) process [22], to a supercritical anti-solvent (SAS) process [23], to a solution-enhanced dispersion by supercritical fluid (SEDS) process [24]. In the SEDS process, supercritical CO_2_ and a solution of chemical compounds in organic solvent are simultaneously sprayed through a co-axial nozzle, where the spraying and dispersion of the initial solution can be significantly enhanced by that of supercritical CO_2_. The moment the drug solution makes contact with supercritical CO_2_, extremely rapid extraction of organic solvent in the drug solution by supercritical CO_2_ occurs, which causes instant drug precipitation and forms particles on a nano/micro-scale [25]. 

In the present study, we attempted to prepare PVM/MA nanoparticles by the SEDS process. A 2^3^ factorial experiment was designed to study these parameters systematically. PVM/MA was first homogeneously dissolved in acetone and then sprayed into supercritical CO_2_ by the SEDS process. PVM/MA before and after the SEDS process was characterized by scanning electron microscopy (SEM), FTIR, TG-DSC, and GC; the corresponding zeta potential was also evaluated.

## 2. Results and Discussion

### 2.1. Particle Size and Particle Size Distribution of PVM/MA Particles

**Figure 1:**
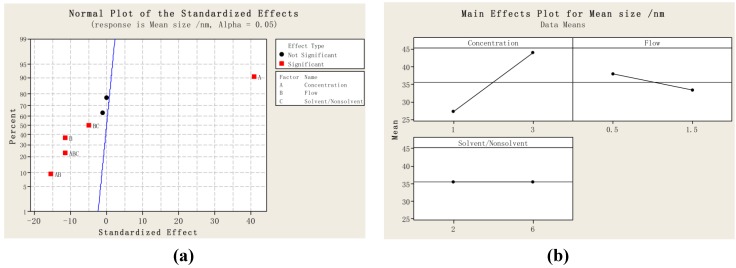

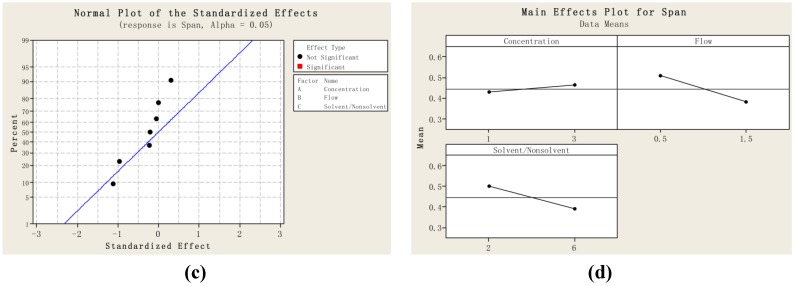
(**a**) Standardized effect of factors on mean size; (**b**) Main effects plot for mean size; (**c**) Standardized effect of factors on the span; and (**d**) Main effects plot for the span. ((A: the concentration of poly-(methyl vinyl ether-co-maleic anhydride) (PVM/MA) (%); B: Flow rate of PVM/MA solution (mL·min^−1^); and C: solvent/nonsolvent (acetone:DCM)).

Table 1 shows the experimental results on mean particle size and particle size distribution, where D10, D50, and D90 are the equivalent volume diameters at 10%, 50%, and 90% of the cumulative volume; and the span is defined as (D90-D10)/D50. A smaller span indicates a narrower particle size distribution. Figure 1 shows (a) the standardized effect of the factors on mean size; (b) the main effects plot for mean size; (c) the standardized effect of the factors on the span; and (d) the interaction plot for the span, which was obtained from the statistical analysis.

**Table 1:** Mean size and particle size distribution of PVM/MA particles prepared by supercritical CO_2_ (SEDS).

Run order	Mean size/nm	D10	D50	D90	SPAN
1	27.6	21.3	27.0	36.0	0.544
2	46.3	34.1	44.8	60.2	0.582
3	26.6	21.3	26.1	32.1	0.414
4	42.0	32.5	42.5	52.0	0.459
5	25.4	20.1	25.1	30.5	0.414
6	52.4	38.1	52.6	64.0	0.492
7	29.7	24.6	29.6	34.6	0.337
8	34.9	29.0	35.3	40.2	0.317
9	35.3	29.0	35.1	43.4	0.418
10	36.4	28.3	36.5	45.2	0.463
11	35.1	22.6	35.0	48.3	0.734

As shown in Figure 1a, factor A (the concentration of PVM/MA) has the most significant effect on mean particle size (*p* < 0.002). Factor B also has a significant effect on mean particle size (*p* < 0.03). Furthermore, there are strong interactions between the factors, including AB (*p* < 0.02), ABC (*p* < 0.03), and BC (*p* < 0.05). Figure 1b shows the main effects plot for mean size; in the range of the parameters studied, the particle size decreased with a decreasing concentration of PVM/MA, and an increasing flow rate. Figure 1c shows that no factor has a significant effect on the span (*p* > 0.05), which is also confirmed in Figure 1d; in the range of the parameters studied, the increases or decreases in any one of the factors did not change the span significantly. From the above results, the effect of the concentration of PVM/MA was found to be the most dominant in the results regarding particle size. This is consistent with the general acceptance that the concentration of the initial solution is the factor that has the greatest influence on the particle size. It is because that when the concentration of PVM/MA becomes higher, the time for crystal growth increases, thus giving larger particles; and an increase in concentration causes an increase in the viscosity and surface tension of the solution, resulting in the formation of larger primary droplets, which consequently producing larger particles [26,27]. In Kalani’s review [28], the effect of concentration has been comprehensively interpreted. The initial concentration of the solution actually has two opposite effects on particle size. On the one hand, a higher concentration will generate a higher supersaturation and faster nucleation, thus reducing the particle size and particle size distribution; on the other hand, a higher concentration will cause higher condensation and increase the particle size and widen particle size distribution due to the longer time for crystal growth. For the effect of flow, it is generally accepted that increasing the ratio of CO_2_ flow rate to the organic solution flow rate reduces the particle size [28]; since the flow rate of CO_2_ was kept in constant, the increasing in flow rate of organic solution would increase the particle size. However, the results of this study indicate that increasing the flow rate of organic solution decreased the particle size and narrowed the particle size distribution. This can be explained that higher solution flow rates will enhance the atomization in the nozzle and generate a smaller droplets size, thus forming a smaller particle size and a narrower particle size distribution; on the contrary, when low solution flow rates are used, a mixture of large and small particles is obtained [29,30]. For the effect of solvent/nonsolvent ratio, different phenomenon was observed that when using the SEDS process to precipitate puerarin, the effect of the nonsolvent/solvent (DCM/ethanol) ratio was found to be dominant in the results regarding particle size, increasing the nonsolvent content of the puerarin solution decreased the particle size significantly [31]. This difference can be explained in terms of nucleation and growth processes. In the precipitation of puerarin, since the solvent of ethanol has a relatively low solubility in supercritical CO_2_ [32], the mixing of DCM in ethanol would greatly increase the speed of extraction of ethanol by supercritical CO_2_ [33], and thus generating a much faster nucleation process, which would be the prevailing mechanism for precipitation of puerarin. While in the precipitation of PVM/MA, since both acetone and DCM have a high solubility in supercritical CO_2_ and would be extracted by the supercritical CO_2_ quickly [33,34,35], the mixing of DCM in acetone could not generate a faster nucleation process, and thus the growth process would be the prevailing mechanism for precipitation of PVM/MA.

### 2.2. Physicochemical Characterization of PVM/MA Nanoparticles

**Figure 2:**
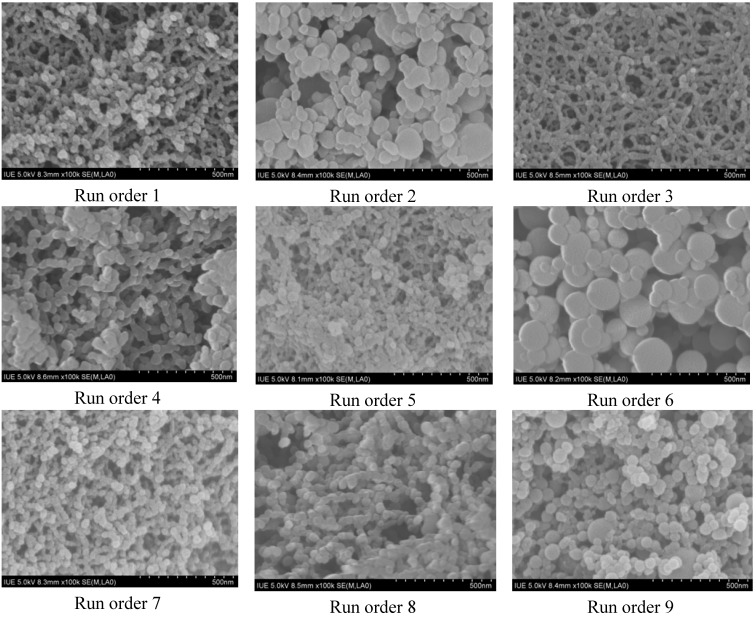
SEM photographs of PVM/MA nanoparticles obtained in different run orders as shown in Table 1

Figure 2 shows the SEM photographs of samples obtained in the different run orders given in Table 1. Following SEDS processing, the PVM/MA nanoparticles are uniform in size, with a shelled peanut-like morphology. The SEM images of Figure 2 provide a visualization of the quantitative data analysis displayed in Figure 1, showing that the SEDS processing parameters have a significant influence on the morphology, particle size, and size distribution. 

After SEDS processing, the original PVM/MA powders were micronized into nanoparticles; the effect of the concentration of PVM/MA was found to be dominant in the results regarding particle size. In addition, from the SEM images, it was found that increasing the PVM/MA concentration tended to increase the width of the particle, while increasing the flow rate tended to increase the length of the particle and create a shelled peanut-like morphology. An increase in the degree of saturation, and a decrease in the PVM/MA concentration or flow rate, generated smaller particles that tended to be more spherical. 

**Figure 3:**
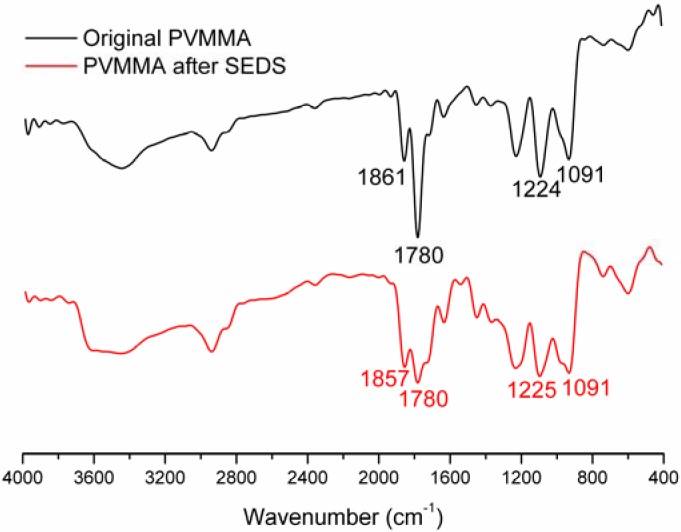
FTIR spectra of original PVM/MA and PVM/MA nanoparticles prepared under the optimized operating parameters (concentration: 1% wt/v, flow: 1.5 mL min^−1^, solvent/nonsolvent: 6)

For further physicochemical characterization, the samples prepared under the optimized operating parameters (Run order 7: concentration: 1% wt/v, flow: 1.5 mL min^−1^, solvent/nonsolvent: 6) were used. As shown in Figure 3, the major peak at 1780 cm^−1^ of the FTIR spectra for PVM/MA, was due to the stretching vibration of the carbonyl group and can be used to identify PVM/MA. Apart from that, no significant difference and no new peaks were found in the FTIR spectra of the original PVM/MA and PVM/MA nanoparticles; that is to say, no chemical reaction occurred in the SEDS process. This all demonstrates that the SEDS process is a typical physical process and this process is favorable for drugs, since chemical interaction may alter the properties of drugs and reduce their effectiveness.

The zeta potential measurement showed that the absolute value of zeta potential of the PVM/MA nanoparticles was larger than 40 mV, which indicates that the PVM/MA nanoparticles can offer good stability in aqueous suspension. The results of TG-DSC measurements (as shown in supplementary material) reveal there was almost no difference between the TG-DSC curves of the original PVM/MA and PVM/MA nanoparticles, indicating that the SEDS process has a negligible effect on the coating polymer’s thermostability.

In this present study, acetone was used as a solvent of the biodegradable polymer. It is essential to determine the acetone residue in the final product. Without any further treatment, the results of head-space GC analysis showed that the acetone residue in the PVM/MA nanoparticles was 0.002% (wt/wt), which is much lower than the maximum acceptable limit (0.5% wt/wt) of *the Pharmacopeia of People’s Republic of China (2010)*. This result reveals that the SEDS process could generate drug-loaded polymer particles with little organic residue and avoid the subsequent tedious removal process, which would be resource-intensive, especially in the context of large-scale production.

## 3. Experimental Section 

### 3.1. Materials

PVM/MA, with a gross molecular weight of 2,160 kDa, was purchased from Sigma-Aldrich. Acetone (AR) was purchased from the Sinopharm Chemical Reagent Co., Ltd. (Beijing, China). CO_2_ with a purity of 99.9% was provided by the Rihong Industrial Development Co., Ltd. (Xiamen, China). Other reagents were all of analytical reagent grade. All the chemicals were used as received without further purification. 

### 3.2. Methods 

#### 3.2.1. Nanonization of PVM/MA by the SEDS Process

PVM/MA was dissolved in a certain volume of acetone, and an organic nonsolvent of PVM/MA, DCM, was added to the solution of PVM/MA in acetone to obtain a homogeneous solution with a higher saturation ratio. 

**Figure 4:**
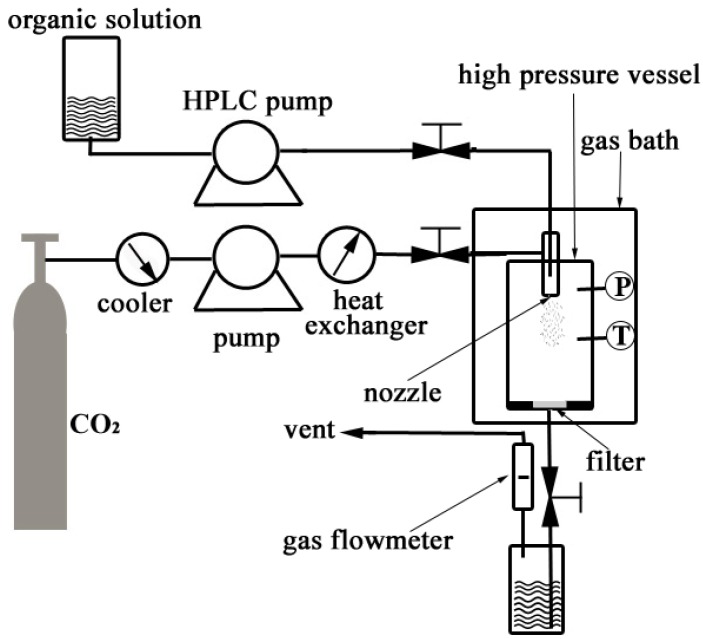
Schematic diagram of the apparatus for the SEDS process.

Figure 4 shows a schematic diagram of the apparatus used for the SEDS process, which consists of three major components: a CO_2_ supply system, an organic solution delivery system, and a high pressure vessel with a volume of 1000 mL.

In the SEDS process, the CO_2_ fed from a CO_2_ cylinder was cooled down to around 0 °C by a cooler in order to ensure the liquefaction of the gas and also to prevent cavitations. A high pressure meter pump (2J-X8/32, Hangzhou Zhijiang Petrochemical Equipment CO., LTD., China) was then used to deliver liquefied CO_2_ to the high pressure vessel (1L, Nantong Zhongjing Supercritical Equipment CO., LTD., China), and a heat exchanger was used to preheat the liquefied CO_2_ to the desired operating temperature after it left the pump head. The high pressure vessel was incubated in a gas bath to keep the temperature constant during the experiment. When the desired pressure in the vessel was reached, a steady flow of CO_2_ was maintained, and the system pressure was controlled by adjusting a downstream valve and monitored by a pressure gauge to keep the pressure constant. When the desired pressure and temperature were stabilized, the PVM/MA solution was delivered into the high pressure vessel through a stainless steel coaxial nozzle (inner diameter 0.1 mm, outer diameter 0.5 mm) using an HPLC pump (310SFT01, Scientific Systems, Inc., State College, PA, USA). During the process, the pressure, temperature, and flow rate of CO_2_ were kept at 12 MPa, 33 °C, and 1705 standard liters per hour (NL h^−1^), respectively. When the spraying was finished, fresh CO_2_ was used to wash the products for about 30 minutes in order to remove the residual organic solvent. During the process of washing, the system operating conditions were maintained as described before. After washing, the high pressure vessel was slowly depressurized and the products were collected for characterization. 

#### 3.2.2. Experimental Design

By analyzing the SEDS process, the key variables were identified as follows: ratio of nonsolvent/solvent, PVM/MA concentration, and flow rate of the solution. To investigate the influence and significance of the three variables and their interactions on the surface morphology and particle size, a 2^3^ factorial experiment was designed and conducted, as shown in Table 2. The levels of the variables were determined on the basis of pilot experiments. In addition, three experiments were carried out at the central point to estimate the variances in the process. Analysis of variances was applied to the experimental data using the MINITAB software version 15. Additional experiments were carried out to confirm the experimental conditions that generated superfine particles identified from the above factorial experiments.

**Table 2:** Experiment of the factorial design

Run order	Center point	Blocks	Factor A concentration/% wt/v	Factor B Flow/mL min^−1^	Factor C Solvent/nonsolvent
1	1	1	1	0.5	2
2	1	1	3	0.5	2
3	1	1	1	1.5	2
4	1	1	3	1.5	2
5	1	1	1	0.5	6
6	1	1	3	0.5	6
7	1	1	1	1.5	6
8	1	1	3	1.5	6
9	1	1	2	1.0	4
10	1	1	2	1.0	4
11	1	1	2	1.0	4

#### 3.2.3. Physicochemical Characterization of PVM/MA Nanoparticles 

The surface morphology of PVM/MA nanoparticles was investigated using SEM equipment (S-4800, Hitachi, Ltd., Tokyo, Japan). Before observation, the sample was adhered onto a piece of carbon film that was self-adhered on an aluminum stub and then coated with a thin layer of gold. The particle size and particle size distribution were measured by analyzing the micrographs with Image-ProPlus 6.0 and Origin 8.5. After factorial experiments, the samples prepared under the optimized operating parameters were used for further physicochemical characterization. In the FTIR analysis, sample powders and KBr were mixed gently to prepare the KBr pellets. FTIR spectra of PVM/MA before the SEDS process and PVM/MA nanoparticles prepared by the SEDS process were obtained with an FTIR spectrometer (8400S, Hitachi, Ltd., Tokyo, Japan) in transmission mode, with the wavenumber range of 400–4000 cm^−1^ at a resolution of 4 cm^−1^. In the Zeta potential measurement, five milligram samples were placed in a centrifuge tube, washed with 15 mL of deionized water, and dispersed by ultrasound for 30 min. After the sample suspension cooled to room temperature, the zeta potential was measured using the Malvern Zen 3600 Zetasizer. In the analysis of TG-DSC, ten milligram samples were uniformly dispersed in the bottom of an Al_2_O_3_ crucible and TG-DSC measurement was performed in a nitrogen atmosphere using a Netzsch STA 449C (Netzsch Instruments, Burlington, Germany) at a heating rate of 10 °C/min over a temperature range of 20–800 °C GC (6850A, Agilent Technologies Inc., Santa Clara, CA, USA) was also utilized to evaluate the acetone residual in the PVM/MA nanoparticles. Samples of approximately 500 mg, without any further treatment, were accurately weighed and the analysis was performed using the static head-space method.

## 4. Conclusions 

PVM/MA nanoparticles with a mean size ranging from 25.4 nm to 52.4 nm were successfully prepared by the SEDS process and the significance of the relative operating parameters was studied systematically. The effect of the concentration of PVM/MA was found to be dominant in the results regarding particle size. Decreasing the initial solution concentration can significantly reduce the obtained particle size (*p* < 0.05); while increasing the flow rate tended to increase the length of the particle and create a shelled peanut-like morphology. The results in this study demonstrated that the SEDS process was a typical physical process and effective in producing smaller fine particles of biodegradable polymer and reducing the usage of CO_2_. It can be expected that the drug-loaded polymer nanoparticles prepared by the SEDS process might have a great potential in the application of drug delivery systems.

## Supplementary Materials

Supplementary File 1
