# Supplementary material for: Preparation of Poly-(Methyl vinyl ether-co-maleic Anhydride) Nanoparticles by Solution-Enhanced Dispersion by Supercritical CO2
Source: Materials (Basel). 2012 Oct 10;5(10):1841–52. doi: 10.3390/ma5101841 (PMC5449034; doi:10.3390/ma5101841)
Supplement: Supplementary File 1 [file materials-05-01841-s001.pdf]

Supplementary Information

Figure S1. TG-DSC curves of (**a**) the original PVM/MA; and (**b**) the PVM/MA nanoparticles prepared under the optimized operating parameters (concentration: 1% wt/v; flow: 1.5 mL min^{-1}; solvent/nonsolvent: 6).

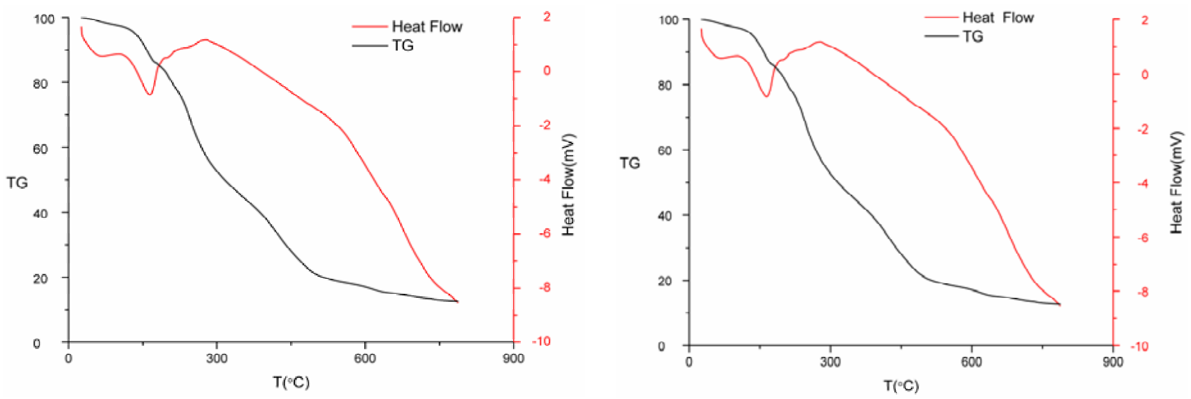

(a) **(b)**

© 2012 by the authors; licensee MDPI, Basel, Switzerland. This article is an open access article distributed under the terms and conditions of the Creative Commons Attribution license (http://creativecommons.org/licenses/by/3.0/).
